# Study protocol for artificial intelligence-assisted sponge cytology as pre-endoscopy screening for early esophegeal squmaous epithelial lesions in China

**DOI:** 10.1186/s12885-022-10220-3

**Published:** 2022-10-28

**Authors:** Yadong Feng, Bin Wang, Liang Pan, Bin Yao, Bin Deng, Yan Liang, Yongzhen Sun, Juncai Zang, Xinyi Xu, Jie Song, Mengjie Li, Guangpeng Xu, Kai Zhao, Cui-E. Cheng, Ruihua Shi

**Affiliations:** 1 Department of Gastroenterology, School of Medicine, Zhongda Hospital Southeast University, 87 Dingjiaqiao Road, Nanjing, 210009 China; 2 Department of Gastroenterology, Changshu No.2 People’s Hospital, the Affiliated Changshu Hospital of Xuzhou Medical University, 18 Taishan Road, Suzhou, 215500 China; 3 Department of Gastroenterology, Jintan Hospital Affiliated to Jiangsu University, 500 Jintan Avenue, Changzhou, 213200 China; 4 Southeast University, 2 Sipailou, Nanjing, 210096 China; 5 Department of Gastroenterology, Affiliated Hospital of Yangzhou University, 386 Hanjiang Middle Road, Yangzhou, 225001 China; 6 Froeasy Technology Development CO., LTD, Red Maple Park of Technological Industry, C1 Building, Nanjing, 210046 China

**Keywords:** Esophageal squmaous cell carcinoma, Artificial intelligence, Cytology, Pre-endoscopy, Screening

## Abstract

**Background:**

Endoscopic screening is the widely accepted screening strategy for esophageal squmaous cell carcinoma (ESCC). However, massive endoscopic screening is expensive and not cost-efficient, and novel pre-endoscopy detection used as a preliminary screening method arouses new concerns. We are planning to launch an artificial intelligence (AI) assisted sponge cytology for detecting esophageal squmaous high-grade intraepithelial neoplasia (HGIN) and above lesions. The aim of this trail is to investigate the efficiency of AI-assisted sponge cytology in population-based screening of early esophageal squmaous epithelial lesions.

**Methods:**

The study will be prospectively conducted in five regions with a high prevalence of ESCC. AI-assisted sponge cytology and endoscopic examination will be sequentially performed. Based on our previous data, at least 864 patients with esophageal HGIN and above lesions are needed to achieve enough statistical power. And, a calculated 112,500 individuals with high risks of ESCC will be recruited. In the first stage, each 24,000 participants who meet the inclusion criteria will be recruited on a voluntary basis. Setting pathological results as standard reference, diagnostic threshold and according performance of AI-assisted detection will be evaluated. A prediction model will be constructed by co-analyzing cytological results and relevant risk factors. Then, an external validation cohort will be used for validation of the model efficiency. Also, cost-efficiency analysis will be performed. This study protocol was registered on chineseclinicaltrial.gov (ChiCTR1900028524).

**Discussion:**

Our study will determine whether this AI-assisted sponge cytology can be used as an effective pre-endoscopy detection tool for large-scale screening for ESCC in high-risk areas.

**Supplementary Information:**

The online version contains supplementary material available at 10.1186/s12885-022-10220-3.

## Background

Esophageal cancer (EC) is the sixth leading cause of mortality and the seventh cause of morbidity among all cancers [1]. Esophageal squamous cell carcinoma (ESCC) is the predominant histological subtype of EC, and accounts for about 85% cases of EC [1, 2]. The overall 5-year survival rate of early ESCC is over 90% [3], but is less than 30% in advanced stage of disease [4]. This great gap in survival status indicates that it is important to adopt an early detection strategy to enable early diagnosis in areas with high risks of ESCC.

### Current status of ESCC screening

High risk factors of ESCC have been proposed, including family history of ESCC, heavy alcohol drinking, cigarette smoking, poor nutritional status, salted food preference, high temperature food preference, chemical carcinogen exposure and history of SCC of the head and neck or upper respiratory tract, et al. [5, 6].

Since critical risk factors have not been revealed, it is difficult to make significant breakthroughs in primary prevention of ESCC. So, secondary preventions aiming at early detection of precancerous lesions and intervention to prevent the disease progression have been considered appropriate strategies to focus on for ESCC control. Due to high sensitivity and accuracy, endoscopic screening has been widely used in population-based screening in China. A strategy of risk-stratified endoscopic screening has been proposed [7]. Endoscopic detection for ESCC and its precursor lesions is applied in 40–69 years aged permanent residents with one or more risks. However, the implementation of endoscopic screening for early ESCC and high-grade intraepithelial neoplasia (HGIN) is very costly because the demand of endoscopic screening from eligible individuals in high-risk areas of China is high. In addition, the incidence of ESCC, even in those regions with a high prevalence, is relatively low [5]. Hence, to develop a novel pre-endoscopy screening method will promote screening effectiveness and make it possible for large-population screening.

Although cytology has been used as pre-endoscopy detection, its role is limited due to poor diagnostic accuracy, low sensitivity and specificity [6, 8]. One main reason is that cytopathologists screen all slides manually for potentially interested lesions and it is not suitable for massive screening [9]. Therefore, the change of diagnostic manner will promote further application of cytological methods in screening of early ESCC and its precursor lesions. Similarly, a rigid diagnostic threshold for such detection should be established [10].

### Artificial intelligence (AI)-assisted cytological detection

We have introduced a novel sponge device (Shikang I®/Eso Heal I™, Froeasy Tech Co., Nanjing, China) for esophageal epithelial sampling [11, 12]. Also, we have reported our pilot performances [11, 12] of AI-assisted cytological detection for early ESCC and its precursor lesions by using a novel platform from Froeasy Tech Co., which is available at http://pms.n-ecdc.com/. Esophageal squmaous epithelial lesions can be classified into five levels, including normal squmaous cells, atypical squmaous cells (ASC), low-grade squamous intraepithelial lesion (LSIL), high-grade squamous intraepithelial lesion (HSIL) and squmaous cell carcinoma (SCC). The AI-assisted diagnosis has shown a diagnostic accuracy comparable to that by expert cytopathologists [11, 12].

We have reported a rapid work scheme for esophageal sponge cytology for detecting early ESCC [11]. The feasibility of this AI-based detection has been preliminarily established, as reflected by significant reduction of numbers of endoscopic examination for detecting esophageal lesions with clinical significance [11, 12]. In addition, the cost of community-based screening was significantly reduced by using this AI-assisted sponge cytology as pre-endoscopy screening [12].

Currently, role of AI-assisted sponge cytology in large-scale screening for esophageal HGIN and above lesions has not been confirmed. We assume that population-based screening can be facilitated by this AI-assisted cytology. Also, the cost-effectiveness of population-based screening may be improved.

### Study design

The project will be prospectively executed in five regions with a high prevalence of ESCC, including Huaian, Taizhou, Yangzhou, Suqian and Yancheng, in Jiangsu Province, China. Participants will be free of charge. Our primary objective is to investigate the efficiency of AI-assisted sponge cytology in population-based screening for esophageal HGIN and early ESCC. Secondary objectives include: (1) to establish the diagnostic threshold (i.e. ASC, LSIL [10]) in such cytological detection; (2) to construct a predicting model identifying individuals for further endoscopy examination by co-analyzing cytological features and relevant risk factors; (3) to analyze cost-effectiveness of this AI-assisted cytology in large-scale screening.

**Fig. 1:**
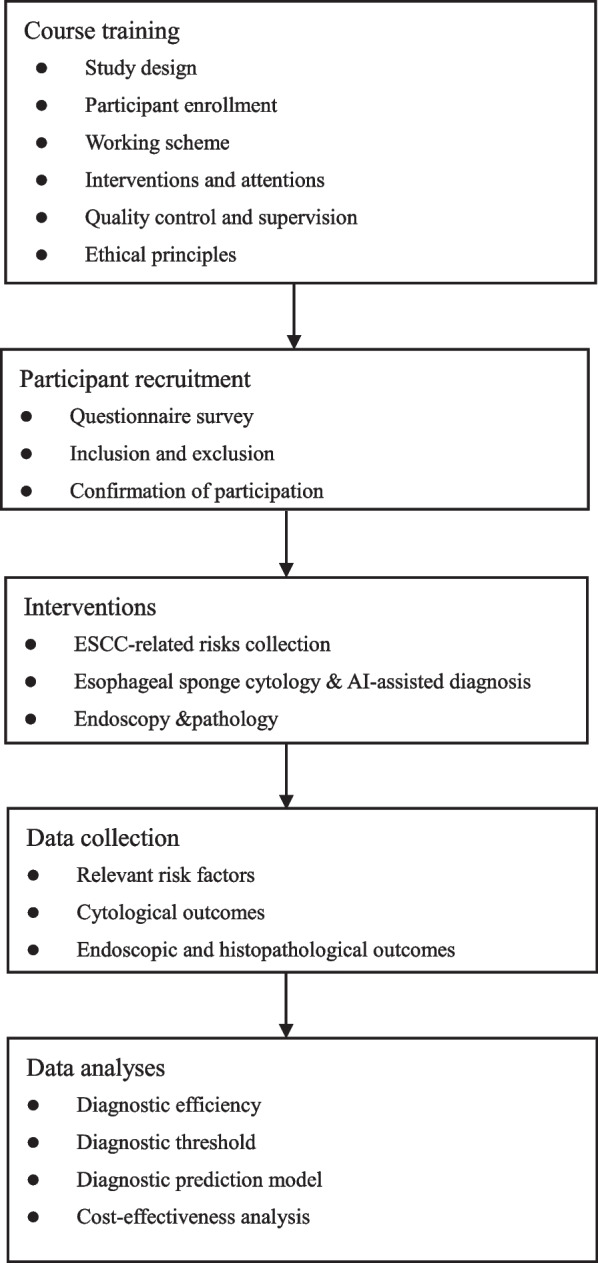
Flowchart of this study. ESCC: esophageal squmaous cell carcinoma

This is a two-stage, 5-year-period project. The flowchart of this study is listed in Fig. 1. The procedures include ESCC-related questionnaire collection, esophageal sponge sampling followed by AI-assisted diagnosis, and endoscopic examination and histopathlogical evaluation. Histopathological results are set as standard of diagnosis of esophageal HGIN and ESCC. In the first stage, population-based esophageal sponge cytology and endoscopic examination will be sequentially performed in four years. Data regarding ESCC-related risks, cytological outcomes, and endoscopic and pathological findings will be collected. Diagnostic efficiency and diagnostic threshold of this AI-assisted cytological diagnosis will be evaluated. By co-analyzing cytological outcomes and relevant risks, a prediction model will be constructed to identify individuals with histopathologically confirmed esophageal HGIN and above lesions. In the second stage, an external individual cohort will be enrolled. The prediction model will be validated, and the diagnostic performance will be assessed. Also, cost-efficiency analysis will be performed in two stages. Our working schedule and relevant results of this study are shown in Table 1.

**Table 1:** Working schedule of this study

Time length	Activities	Expected stage outcomes
1^st^ year	• Project announcement, planning and preparation • Course training and project coordination • Participant recruitment • Cytological screening &endoscopy • Data collection and transfer • Scientific visit and supervision	♦ Project initiation ♦ Screening 4,000 participants from each site
2^nd^ year	• Participant recruitment • Cytological screening &endoscopy • Data collection and transfer • Scientific visit and supervision	♦ Screening 8,000 participants from each site
3^rd^ year	• Participant recruitment • Cytological screening &endoscopy • Data collection • Scientific visit and supervision	♦Screening 8,000 participants from each site ♦ Interim report ♦ Preparation of the first publication
4^th^ year	• Participant recruitment • Cytological screening &endoscopy • Data collection • Data verification and analysis • Scientific conference	♦ Screening 4,000 participants from each site ♦ Data analysis assessment of diagnostic efficiency construction of prediction model cost-efficiency analysis
5^th^ year	• Participant recruitment • Cytological screening &endoscopy • Data analysis and archive • Project summary	♦ Screening 2,000 participants from each site ♦ External validation ♦ Publication of results ♦ Dissemination of results to public health agencies

Some key procedures, including course training, participant recruitment, esophageal sponge cytology and endoscopy, will be used in this study, and the details are listed as follows.

1. Course training
  Courses about the program will be given to train community health workers, endoscopists and nurses prior to participant recruitment. Contents of training are as follows: (1) nature, purpose, ethical principles, study design and working scheme of this program; (2) questionnaire survey as a structured sheet of ESCC-related risks; (3) notices in esophageal sponge ingestion and sampling; (4) notices in endoscopic examination; and (5) quality control in cytological sampling and endoscopic biopsy.
2. Participant recruitment
  Recruitment announcements will specify the eligibility criteria, place and time of the screening. Information regarding this program will be spread through several approaches, including radio, television and paper announcements, public banners, free-accessible apps of hospitals and community health sectors, short messages by public health platform, and so on. Community health workers will also be specially trained to recruit participants. The free nature of this project will be emphasized.
  Potential eligible participants will be identified by a questionnaire survey for ESCC-related risks among 40–69-year-old permanent residents. Inclusion and exclusion criteria are listed as follows. Those who are deemed high risky of ESCC and voluntary to participate in this program will be recruited. Those who meet one or more of following items will be excluded: (1) < 40 years or ≥ 70 years; (2) pregnant women at the screening consultation; (3) prior history of esophageal cancer, esophageal stenosis, esophagectomy, esophageal varies or mental disorder; (4) positive for human immunodeficiency virus; (5) insufficient health to participate in this screening; (6) refusal to participate in this screening; (7) unwilling to undergo capsule sponge ingestion and/or endoscopy.
  Written consent is mandatorily required from each participant, and a copy of electronic record will be reserved. Subject confidentiality will be ensured by utilizing subject identification code numbers. ESCC-related risks from each individual will be collected.
3. Esophageal sponge cytology and diagnosis
  Esophageal epithelial specimens will be collected by sponge device ingestion. And, cytological diagnosis will be performed in an AI-assisted manner. Key steps are listed as follows. (1) Esophageal epithelial specimens will be collected by an esophageal sponge device swallowed into the stomach [11, 12]. (2) The mesh along with specimens will be retrieved out of the mouth gently. Retrieval of sponge mesh will be attempted by each participant, and a standby health worker will be ready to provide help anytime necessary. The mesh containing specimens will be kept in cell preservation solution (Froeasy Tech Co., Chinese inventory patent is pending, ZL201810313809.8). (3)Specimens will be processed by liquid-based thin layer cell preparation technique. By using a sedimentation technology, 120 slides will be prepared for each specimen [11]. Feulgen-Eosin staining (Chinese invention patent, ZL 201,710,732,464.5) will be adopted. (4) All slides will be subjected to the AI-based diagnosis system. An automated squmaous epithelial cell classification will be given by AI-assisted diagnosis platform, and ASC and above cells will be automatically annotated by this system [11]. (5) Cytological outcomes of ASC and above will be confirmed by three expert cytopathologists independently [11]. As listed in the Supplementary material, specimen processing, slides preparation and staining, and AI-assisted diagnosis will be performed in a batch processing strategy.
4. Endoscopy
  Endoscopy will be used to detect histopathologically confirmed esophageal lesions. An early endoscopy, which is completed within five working days, is recommended, but any time interval no longer than twenty-working-day is also acceptable. Participants who did not undergo endoscopy examination will be reminded by phone calls once every week; failing to undergo scheduled endoscopy examination within the time-frame will be excluded from this trial. Endoscopic examination will be performed under shallow sedation (midazolam, 0.1 mg/kg, ivggt; fentanyl, 1 μg/kg, ivggt) by competent (more than 5 years of experience in endoscopic procedures) or expert (more than 10 years of experience in endoscopic procedures) endoscopists at each study site. White light imaging and Lugol iodine staining will be sequentially performed to inspect the whole length of the esophagus and to evaluate suspected lesions. At least one biopsy will be taken from each suspected region. A normal solution of 5% sodium thiosulfate will be sprayed for reducing adverse events relevant to Lugol iodine staining. Histological diagnosis of esophageal biopsy will be classified into normal squamous epithelia, esophagitis, squamous cell hyperplasia, low-grade intraepithelial neoplasia (LGIN), HGIN, and ESCC.
5. Data analysis
  Diagnostic accuracy of AI-assisted cytology for esophageal HGIN and early ESCC will be evaluated and compared to that of endoscopic detection. Diagnostic performances will be compared between different thresholds (ASC and above vs. LSIL and above). A prediction model for esophageal HGIN and early ESCC will be built based on co-analyzing with ESCC-related risks.
  Cost data will be collected since the start of the study regarding personnel remuneration, training and equipment costs. Further data will be collected regarding the time and cost required for each step of the project. The Markov model will be used for cost-effectiveness analysis.
6. Diagnostic standard
  Based on our previous work [11, 12], cases diagnosed with ASC and above level lesion by AI system will be manually confirmed by two expert cytopathologists according to the Bethesda system [13]. Histopathological results from biopsies will be set as the gold standard for diagnosis of esophageal HGIN and early ESCC. Two expert pathologists who are masked of cytology results will provide consensual diagnosis.
7. Supervision and quality control

Principal investigator, co-investigators or responsible researchers will visit each study site once every six months to give professional and scientific opinions for the aim of supervision of this project.

### Endpoints

Primary endpoint is a two-stage estimation of diagnostic efficiency of the AI-assisted cytology by using histopathological diagnosis as reference. Secondary endpoints include: (1) comparison of diagnostic accuracies among AI-assisted cytology, endoscopy and pathology; (2) construction and validation of prediction model based on cytological outcomes and risk factors; (3) assessment of cost-effectiveness of AI-assisted cytology as pre-endoscopy detection.

### Statistical analysis

The diagnostic accuracy, sensitivity, specificity, positive predictive value (PPV), negative predictive value (NPV), positive likelihood ratio (PLR), and negative likelihood ratio (NLR) for each test will be calculated with 95% confidence intervals. The baseline participant characteristics will be categorized in percentages for categorical variables and as mean ± SD (Standard Deviation) for continuous variables. The Chi-square test and Mann–Whitney *U* test will be used for analysis of differences. Multivariate logistic regression will be adopted for identifying significant variables. Relevant variables obtained will be included in the multivariable model. The probabilities of diagnosis of esophageal HGIN and early ESCC will be calculated, and an external validation will be performed to evaluate the calibration and discrimination abilities of the model by decision curve analysis. A receiver operating characteristic curve (ROC) analysis will also be applied. Statistical analyses will be performed using SPSS statistical software for Windows, version 22.0 (SPSS Inc., Chicago, IL, USA) and the statistical software package R Version 4.0.5. A *p* value < 0.05 is considered statistically significant.

### Sample size calculation

According to our previous data, a sensitivity of 90.0% was present in AI-assisted esophageal HGIN and above lesions [11]. Sample size will be estimated by using the formula:

$$\small {n=Z\alpha^2}P(1-P)/\delta^{2}$$

where *n* = required sample size, *P* = anticipated sensitivity. The confidence level is set as 95% in this study, and Zα = the corresponding standard normal deviate 1.96. And, δ = permissible error, which is set as 0.02. Therefore, 864 individuals with esophageal HGIN and above lesions are needed. Taking the drop rate of 20% into account, this number will be 1,080. Since the prevalence of disease is about 1.2–1.5% [5, 14], about 90,000 potential eligible participants are needed. Given a response rate at about 80%, participant recruitment will be about 112,500 individuals with high risks of ESCC. Therefore, we plan to carry out recruitment in 24,000 residents from each of the five regions in the first stage.

Sample size for validation is also calculated by using the abovementioned formula. The anticipated sensitivity, the confidence level, Zα and permissible error are set as 90%, 95% and 1.96 and 0.06, respectively. Therefore, 96 patients with esophageal HGIN and above lesions are needed. Taking the drop rate and the prevalence into account, about 10,000 participants are needed. Hence, 2,000 new individuals will be recruited from each study site in the second stage for the aim of validation of the predicting model.

### Participant privacy, safety and supervision

The investigator will affirm and uphold the principle of the participants’ right to dignity, privacy and health. Information obtained as a result of this study is considered confidential and disclosure to third parties is prohibited. Anonymity of the participants will be guaranteed when presenting the data at scientific meetings or publishing them in scientific journals.

The planned measures for collecting personal data, esophageal sponge cytology, and endoscopic examination and biopsy entail only minimal risks [11, 12]. Potential adverse events include vomiting, throat discomfort, retrosternal pain, abdominal discomfort, iodine allergy, bleeding, potential esophageal injury, and so on. Participants with symptomatic discomfort will undergo clinical observation.

## Discussion

Since ESCC is a rare disease even in regions with an extremely high-risk [5], the cost of broad endoscopic screening project should be carefully evaluated. Recently, how to achieve quality improvement of screening has gained much interest [7, 15]. For the purpose of precise screening, this proposed project is aiming to explore the efficiency of an AI-assisted cytology as pre-endoscopy detection in population-based screening in high-risk areas. As shown in our previous results [11, 12], we believe that this trial will contribute to the establishment of a novel massive screening model for ESCC and similar kinds of diseases.

The project will be prospectively conducted in a real-world setting. According to designed research plan, all participants will undergo AI-assisted sponge cytology and endoscopic examination to minimize disease misclassification. Results of this trial will provide a convinced piece of evidence that how this technology can be integrated in massive screening for ESCC. In addition, the feasibility of this strategy as pre-endoscopy detection can be determined by cost-effectiveness analysis.

We suppose that results from this trial will demonstrate the feasibility of a reliable automated cytological detection for esophageal HGIN and above lesions, and the developed scheme will meet the great amount needs of large-scale screening in high-risk areas. In addition, this strategy will be superior to current endoscopic screening program by presenting a more precise, accurate outcome and a more economical procedure. Furthermore, a prediction model for identifying individuals with high-risks of confirmed lesions will be constructed, and will be prospectively validated. Based on relevant cytological outcomes and exposure of potential risks, this model will represent a higher efficiency than those models by evaluating ESCC-related risks only [16, 17].

Our results will be adopted as a promising complementary to current screening program in large-scale screening of ESCC in regions with a high prevalence. Decreased cost and saved medical sources can be achieved. Also, improved social and economic benefits can be brought up by the technique.

## Supplementary Information

**Additional file 1.**

## Data Availability

All data, including the questionnaire survey, cytological outcomes, endoscopic and histopathological results, will be collected from each study site and transferred to the Clinical Research Center (CRC) at Zhongda Hospital Southeast University. All data will be archived at the CRC for a minimum of 10 years after study termination. Authorized representatives of the sponsor, a competent authority or an ethics committee may require direct access to parts of records for the aim of data verification. The anonymized data from this study will also be uploaded to a public repository (such as Synpase), and will be available upon request to the principal investigator for non-commercial purposes.

## Abbreviations

ESCC: Esophageal squmaous cell carcinoma
AI: Artificial intelligence
HGIN: High-grade intraepithelial neoplasia
EC: Esophageal cancer
ASC: Atypical squmaous cells
LSIL: Low-grade squamous intraepithelial lesion
HSIL: High-grade squamous intraepithelial lesion
SCC: Squmaous cell carcinoma
LGIN: Low-grade intraepithelial neoplasia
PPV: Positive predictive value
NPV: Negative predictive value
PLR: Positive likelihood ratio
NLR: Negative likelihood ratio (NLR)
CRC: Clinical Research Center

## References

[1] Sung H, Ferlay J, Siegel RL (2021). Global Cancer Statistics 2020: GLOBOCAN Estimates of Incidence and Mortality Worldwide for 36 Cancers in 185 Countries. CA Cancer J Clin.

[2] Ferlay J, Colombet M, Soerjomataram I (2019). Estimating the global cancer incidence and mortality in 2018: GLOBOCAN sources and methods. Int J Cancer.

[3] Liu Y, Qian D, Tang B (2021). Feasibility of endoscopic submucosal dissection for early esophageal squamous cell carcinoma with relative indications. Dig Surg.

[4] Zeng H, Zheng R, Guo Y (2015). Cancer survival in China, 2003–2005: a populationbased study. Int J Cancer.

[5] He Z, Liu Z, Liu M (2019). Efficacy of endoscopic screening for esophageal cancer in China (ESECC): design and preliminary results of a populationa-based randomized controlled trial. Gut.

[6] Gao QY, Fang JY (2015). Early esophageal cancer screening in China. Best Pract Res Clin Gastroenterol.

[7] Li H, Ding C, Zeng H (2021). Improved esophageal squamous cell carcinoma screening effectiveness by risk-stratified endoscopic screening: evidence from high-risk areas in China. Cancer Commun (Lond).

[8] Pan QJ, Roth MJ, Guo HQ (2008). Cytologic detection of esophageal squamous cell carcinoma and its precursor lesions using balloon samplers and liquid-based cytology in asymptomatic adults in Llinxian. China Acta Cytol.

[9] Middleton DRS, Mmbaga BT, O'Donovan M (2021). Minimally invasive esophageal sponge cytology sampling is feasible in a Tanzanian community setting. Int J Cancer.

[10] Bao H, Bi H, Zhang X (2020). Artificial intelligence-assisted cytology for detection of cervical intraepithelial neoplasia or invasive cancer: a multicenter, clinical-based, observational study. Gynecol Oncol.

[11] Feng Y, Liang Y, Yao B (2022). A rapid cytological screening as pre-Endoscopy screening for early esophageal squamous cell lesions: a prospective pilot study from a Chinese academic center. Technol Cancer Res Treat.

[12] Gao Y, Xin L, Feng YD (2021). Feasibility and accuracy of artificial intelligence-assisted sponge cytology for community-based esophageal squamous cell carcinoma screening in China. Am J Gastroenterol.

[13] Nayar R, Wilbur DC (2017). The Bethesda system for reporting cervical cytology: a historical perspective. Acta Cytol.

[14] Abnet CC, Arnold M, Wei WQ (2018). Epidemiology of esophageal squamous cell carcinoma. Gastroenterology.

[15] Chen W, Li H, Ren J (2021). A prediction model of esophageal squamous cell carcinoma based on a multicenter screening cohort in rural China. Int J Cancer.

[16] Han J, Wang L, Zhang H, et al. Development and Validation of an Esophageal Squamous Cell Carcinoma Risk Prediction Model for Rural Chinese: Multicenter Cohort Study. Front Oncol. 202; 11:729471. 10.3389/fonc.2021.729471 PMC8435773 34527592

[17] Yang X, Suo C, Zhang T (2021). A nomogram for screening esophageal squamous cell carcinoma based on environmental risk factors in a high-incidence area of China: a population-based case-control study. BMC Cancer.

