# Supplementary material for: Study protocol for artificial intelligence-assisted sponge cytology as pre-endoscopy screening for early esophegeal squmaous epithelial lesions in China
Source: BMC Cancer. 2022 Oct 28;22:1105. doi: 10.1186/s12885-022-10220-3 (PMC9617337; doi:10.1186/s12885-022-10220-3)
Supplement: Supplementary file 1 — Additional file 1. [file 12885_2022_10220_MOESM1_ESM.docx]

Brief description of specimen processing, slides preparation and AI-assisted diagnosis

A two-round of cell enrichment protocol will be adopted [1]. The sponge with retrieved cells will be washed with 60 ml of preservative fluid and then be oscillated for 15 min in each round. Each slide will be prepared by using 1ml of preservative fluid with suspended cells, followed by Feulgen-Eosin staining. AI-assisted diagnosis can be achieved by an automated slides scan. Automated cell enrichment and slide preparation devices (Froeasy Tech Co., Chinese inventory patent is pending, ZL201810259973.5), a rapid staining method (Froeasy Tech Co., Chinese inventory patent, ZL200810028533.5) and 12-panel double charge-coupled device camera equipped scanning machines (Froeasy Tech Co., Chinese inventory patent, ZL201110071788.1 and ZL201110071786.2) will be applied in general practice. A well-trained cytotechnican can handle 600 specimens daily. And, AI-assisted diagnosis of all these specimens will be available in the same day.

Reference

1. Feng Y, Liang Y, Yao B, et al.  A Rapid Cytological Screening as pre-Endoscopy Screening for Early Esophageal Squamous Cell Lesions: A Prospective Pilot Study from a Chinese Academic Center. Technol Cancer Res Treat. 2022 Jan-Dec; 21:15330338211066200.
